# Alzheimer’s Disease–Thrombosis Comorbidity: A Growing Body of Evidence from Patients and Animal Models

**DOI:** 10.3390/cells14141069

**Published:** 2025-07-12

**Authors:** Joanna Koch-Paszkowski, Christopher Sennett, Giordano Pula

**Affiliations:** Biomedical Institute of Multimorbidity, Centre for Biomedicine, Hull York Medical School, University of Hull, Hull HU6 7RX, UK

**Keywords:** Alzheimer’s disease, thrombosis, platelets, dementia

## Abstract

Background/Objectives: A growing body of evidence is amassing in the literature suggesting a correlation between Alzheimer’s disease (AD) and thrombotic vascular complications, which led to the suggestive hypothesis that thrombosis may contribute to AD onset and progression by damaging the neurovasculature and reducing the cerebral blood flow. In turn, low cerebral blood flow is likely to contribute to neurodegeneration by reducing nutrient and oxygen supply and impairing toxic metabolite removal from the brain tissue. Methods: We searched the literature for studies in animal models of AD or patients diagnosed with the disease that reported circulating markers of platelet hyperactivity or hypercoagulation, or histological evidence of brain vascular thrombosis. Results: Platelet hyperactivity and hypercoagulability have been described in multiple animal models of AD, and histological evidence of neurovascular thrombosis has also been reported. Similarly, clinical studies on patients with AD showed circulating markers of platelet hyperactivity and hypercoagulation, or histological evidence of neurovascular thrombosis collected from post-mortem brain tissue samples. Conclusions: Taken together, a convincing picture is emerging that suggests a strong correlation between systemic or neurovascular thrombosis and AD. Nonetheless, a mechanistic role for haemostasis dysregulation and neurovascular damage in the onset or the progression of AD remains to be proven. Future research should focus on this important question in order to clarify the mechanisms underlying AD and identify a treatment for this disease.

## 1. Introduction

It is increasingly clear that the impairment of circulation within the brain contributes to the progression of dementia. The contribution of neurovascular damage to dementia progression has recently attracted increasing attention [1,2]. Alzheimer’s disease (AD) is the most common form of dementia characterised by amyloid plaque deposition and intraneuronal hyperphosphorylated tau tangles. Although vascular dementia (VD) is considered a separate form of dementia, characterised by a reduction in cerebral blood flow (CBF) as the main cause for neurodegeneration, neurovascular symptoms are commonly reported in AD patients. Therefore, the boundaries between VD and AD are becoming less defined [3]. Similarly, cerebral amyloid angiopathy (CAA), which is characterised by amyloid build-up in the vascular wall (instead of the parenchymal plaque deposition typical of AD), is often observed in AD patients [4], which further complicates the distinction between AD and forms of dementia involving the brain vasculature. Neurovascular damage and reduced brain blood supply have been suggested to contribute to neurodegeneration in both AD animal models [5,6,7] and patients [8,9,10,11,12,13]. In parallel, the number of patients showing both amyloid plaque deposition (i.e., the hallmark of AD) and neurovascular impairment has increased significantly [14], with mixed vascular-Alzheimer dementia (MVAD) becoming the most common forms of mixed dementia (22% of all current dementia diagnoses) [15]. Because of their central role in haemostasis, platelets are critical players in thrombosis, which leads to brain hypoperfusion when it occurs in the neurovasculature [16]. Unsurprisingly, the contribution of the key haemostasis regulator platelets to the progression of vascular dementia and AD has been suggested [17,18,19,20]. Notably, high levels of pre-activated circulating platelets have been reported in patients with AD [21,22,23], and platelet deposits have been observed in the brain microvasculature of AD animal models [24] and patients [25].

**Figure 1:**
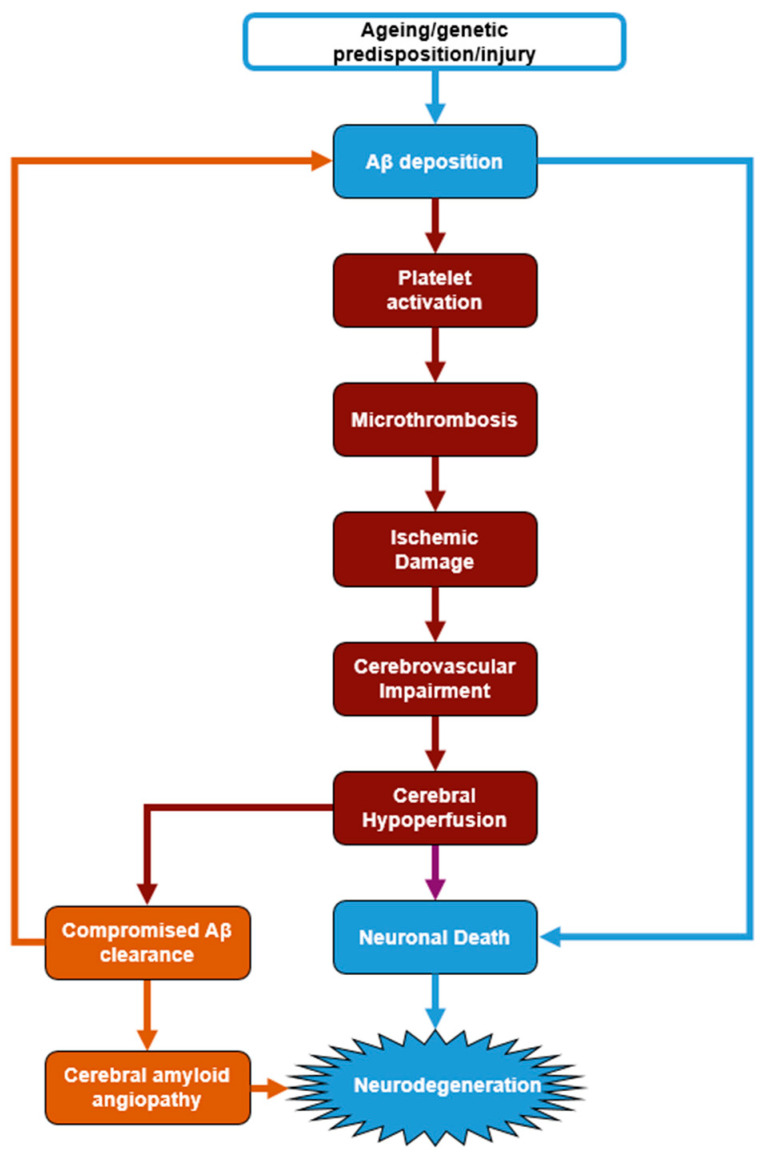
Hypothesis of platelet involvement in AD. Aβ accumulation in the brain during AD triggers platelet activation leading to microthrombosis. The resulting cerebrovascular impairment contributes significantly to disease progression, leading to brain hypoperfusion, inefficient Aβ clearance, and neuronal cell death by nutrient and oxygen starvation. The platelet effects of Aβ are, therefore, proposed to exacerbate neurodegeneration and play a key role in disease progression.

Although proof remains elusive, it is plausible to hypothesise that platelets are mediating the reciprocal effect of amyloid plaque deposition and neurovascular damage in AD, especially in the presence of CAA. The activation of platelets by Aβ is likely to reduce CBF through the induction of microthrombosis, which, in turn, increases plaque formation by reducing Aβ clearance and exacerbates neurodegeneration by reducing the nutrient/oxygen supply and metabolite clearance in the brain (Figure 1).

Platelets are well known for their role in thrombotic vascular conditions and ischemia in the brain vasculature [26] and have been shown to be activated by amyloid β peptide (Aβ) [27,28,29], which is the main component of amyloid plaques in AD. This may cause neurovascular thrombosis in the presence of local or systemically high levels of Aβ. Although several receptors and mechanisms of action have been proposed, oxidative stress has been shown to stimulate platelet apoptosis [30] and is critical for the activation of platelets by Aβ [31,32]. Our group participated in studies showing that Aβ can stimulate platelets by activating nicotinamide adenine dinucleotide phosphate (NADPH) oxidases, leading to protein kinase C and integrin alphaIIb-beta3 stimulation [33], and the scavenger receptor CD36 plays a key role in this response [34]. Nonetheless, this aspect of the physiopathological function of Aβ requires further clarification as multiple other platelet receptors have also been shown to bind directly this molecule, including glycoprotein Ib [34], the fibrinogen receptor integrin alphaIIb-beta3 [35], and the collagen receptor glycoprotein VI [36,37]. Platelets have also been shown to promote the fibrillation of Aβ, which is a prerequisite for the precipitation of this peptide in amyloid plaques, and play a key role in disease progression [36]. Finally, in addition to responding to and modulating the structure of Aβ, platelets are a potential source of this toxic peptide. Their ability to release Aβ has been shown [38], and the biochemical features of AD, including inflammation and neurovascular ischemia, have been shown to modulate their ability to release this molecule [39].

The mechanisms of platelet activation and, in particular, the role of oxidative stress in this phenomenon have been reviewed previously [40]. In this review, we summarise and appraise studies presenting histological evidence of cerebral thrombosis or describing systemic biochemical markers of thrombosis in animal models of dementia or patients diagnosed with dementia. The outlook from this analysis describes a strong correlation between thrombosis and dementia and suggests a potential causative role of neurovascular impairment in the onset and progression of dementia.

## 2. Markers of Platelet Hyperactivity in Animal Models of Dementia

There are several transgenic animal models of AD, with different mutations introduced to mimic the pathophysiology of the disease. They are used to study the aetiology of AD, as well as test potential therapeutic strategies. The models discussed in this review are as follows: The APP23 model expresses human amyloid precursor protein (APP) with the Swedish double mutation (K670N/M671L), causing seven-fold overexpression of APP [41]. 3xTg-AD is a triple-transgenic mouse that expresses the Swedish APP mutation (KM670/671NL), presenilin 1 (PSEN1) M146V mutation, and the microtubule-associated protein tau (MAPT) P301L mutation, and it develops both Aβ plaques and neurofibrillary tangles (NFTs) [42]. APP_SweDI is also a triple-transgenic mouse, with the Swedish (K670N/M671L), Dutch (E693Q), and Iowa (D694N) APP mutations, causing high deposition of Aβ in the brain parenchyma and cerebrovasculature [43]. TgCRND8 carries two APP mutations, Swedish (K670N/M671L) and Indiana (V717F), causing five-fold overexpression of APP [44]. ArcAβ mice overexpress APP with Swedish (K670N/M671L) and Arctic mutations (E693G) [45]. APPPS1 expresses the Swedish APP mutation (K670N/M671L), along with the PSEN1 L166P mutation, with three-fold higher APP expression and deposition of amyloid plaques in the parenchyma but not vessels [46]. Finally, 5xFAD mice have the following five mutations: Swedish APP (K670/M671), Florida APP (I716V), London APP (V717I), PSEN1 (M146L) and PSEN1 (L286V), causing rapid development of amyloid pathology [47].

Multiple studies have shown cerebrovascular alteration in AD. Although the exact role of platelets in AD is still being investigated, it has been shown that platelets can release biologically relevant amounts of Aβ in response to various physiopathological stimuli [39]. Systemic thrombosis and the presence of circulating thrombi have been reported in conditions characterised by neurological deficits [48], but the role of haemostasis dysregulation and vascular damage in AD are still evaluated.

Platelet pre-activation, where circulating platelets show partial activation without being exposed to stimuli, has been shown in APP23 mice [24]. Through flow cytometric analysis, Jarre et al. showed alphaIIb-beta3 integrin activation and P-selectin externalisation (markers of platelet activation) in the platelets of aged APP23 mice without any stimulation. They also showed higher sensitivity to agonists in APP23 mice compared with wild-type controls, including collagen-related peptide, thrombin, adenosine diphosphate (ADP), and the thromboxane A2 (TXA2) analogue (U46619). Additionally, the platelet morphology of aged APP23 platelets was altered after activation with thrombin, which supports the claim of pre-activation of platelets. Further, in vitro, thrombus formation on collagen under physiological flow conditions increased in aged APP23 compared with young APP23 mice and matched wild-type controls. In vivo, they were able to demonstrate alteration of thrombosis in APP23 mice, by observation of reduced thrombus formation and occlusion time after injury of the right carotid induced with FeCl_3_. The tail bleeding time after amputation also decreased after amputation, confirming a pro-thrombotic phenotype in APP23 mice.

Donner et al., on the other hand, evaluated the middle-aged APP23 mice [49]. They showed that although the secretion of dense granules (intracellular membranous bodies storing and releasing biological molecules upon platelet activation) was unchanged, their number increased in APP23 mice compared with an age-matched wild-type. Thrombus formation experiments on collagen under physiological flow showed that thrombus stability decreased in middle-aged APP23 male animals, while, in vivo, an increased duration of thrombus formation after injury (induced with FeCl_3_) was observed [49]. As shown by Jarre et al. [24] and Donner et al. [49], there were differences in platelet reactivity in APP23 mice of different ages. One possible explanation for the differences is the variable level of Aβ accumulation leading to CAA at different ages. Middle-aged animals are pre-CAA and do not have the full phenotype of Aβ accumulation in the brain. Therefore, the platelets are less likely to be in contact with Aβ plaques in the vasculature, which causes a different platelet phenotype. Furthermore, Donner et al. [49] showed higher platelet activation and thrombus formation in male animals compared with female, demonstrating that not only animal age but also sex may influence platelet responsiveness in these animals.

A pro-thrombotic phenotype was also shown in 3xTg-AD mice. The platelets from mice showed enhanced platelet adhesion to collagen, von Willebrand factor, and fibrinogen in static conditions compared with the wild-type controls [50]. Additionally, in vitro thrombus formation under physiological flow conditions on collagen increased in 3xTg-AD compared with wild-type mice. However, aggregation upon stimulation with thrombin receptor-activating peptide 4 (TRAP4), convulxin (agonist of glycoprotein VI), U46619 (TXA2 analogue), and Aβ peptides was unchanged in 3xTg-AD compared to wild-type mice. Platelet hyperactivity or hypercoagulation markers in mouse models of AD are summarised in Table 1.

## 3. Histological Markers of Thrombosis in Animal Models of Dementia

The neurohistological markers of AD are Aβ plaques and NFTs. Histological alterations related to thrombosis are also reported in the brains of AD models. Although AD platelets infused in vivo were shown to concentrate in blood vessels and not penetrate the parenchyma in post-mortem analysis [25], and ex vivo findings showed that the platelets can, indeed, disturb the blood vessel wall. By using ex vivo organoid cultures from healthy wild-type mice, Kniewallner et al. showed the toxic effects of AD platelets on the cerebrovascular system [18]. The brains of wild-type mice were infused with tagged platelets from APP_SweDI mice [18]. The platelets penetrated through cortical brain vessels into the brain parenchyma within two weeks, and the damaged vessels showed increased levels of Aβ [18]. Even in wild-type mice, with induced thrombosis, the level of Aβ was detected in the affected blood vessels [54]. This was not observed in thrombocytopenic mice, showing the direct link between Aβ and platelets [54].

Fibrinogen is a plasma protein that plays a central role in the bridging of platelets during the formation of thrombi and in the thrombin-dependent proteolytic formation of fibrin that stabilises thrombi in the circulation. The co-localisation of fibrinogen chains α, β, and γ with Aβ_40_ in cerebral vessel walls and brain cortex was shown in APP23 mice [51]. The effect was amplified when APP23 mice underwent a surgical procedure to induce chronic cerebral hypoperfusion by the operation) [51]. Fibrinogen accumulation with Aβ plaques was also shown in TgCRND8 mice [52,55], and it has also been shown to increase with age [55]. The localisation of Aβ plaques and fibrinogen also coincided with areas of dystrophic neurites [55]. Additionally, it has been shown that aged TgCRND8 mice exhibit slower clearance of fibrinogen from the brain compared with young TgCRND8 mice and wild-type controls [52]. The reduced clearance explains the fibrinogen accumulation in the brain, despite normal fibrinogen levels in the blood and normal coagulation times (compared with wild-type controls).

The influence of fibrinogen on Aβ accumulation was confirmed by the reduction in fibrinogen pharmacologically or genetically [52]. In mice with lower levels of fibrinogen, the area of Aβ plaques was also reduced [52]. Additionally, genetically altered mice with lower fibrinogen levels expressed reduced areas of dystrophic neurites [55]. Moreover, the treatment with RU-505—an inhibitor of fibrinogen and Aβ interaction—reversed the prothrombotic phenotype and improved cognitive function in Tg6799 mice [53].

## 4. Live Imaging Evidence of Brain Hypoperfusion in Animal Models of AD

Optical in vivo imaging methods, Doppler optical coherence tomography, and spatial frequency-domain imaging were used to assess Aβ plaques and vascular changes in 3xTg-AD mice [56], which highlighted that brain perfusion and oxygenation were reduced in 3xTg-AD, while the vessel volume compared with age-matched controls was reduced. The lower density of vessels in 3xTg-AD mice has been confirmed by immunohistochemistry.

Reduced CBF in the cortex has also been observed in arcAβ mice [45], measured by arterial spin labelling-magnetic resonance imaging (ASL-MRI) [57]. Additionally, photoacoustic tomography has been used to assess brain oxygenation, showing a reduced cerebral metabolic rate of oxygen in transgenic mice. A comparison study performed by Maier et al. [58] has shown a correlation between in vivo brain perfusion and Aβ plaque accumulation in the brain vasculature. Perfusion was measured by ASL-MRI and [^15^O]H_2_O positron emission tomography (PET), whilst the load of Aβ was measured by ^11^C-labelled Pittsburgh compound-B (PIB). The study was conducted in APPPS1 mice, which do not develop CAA and have Aβ plaques located mainly in the parenchyma, and APP23 mice, which do develop CAA. Reduced regional CBF (rCBF) was shown in APP23 mice, correlating with an increased accumulation of Aβ in vessels. However, in APPPS1 mice, there were no changes to the rCBF, confirming that CAA is associated with a reduction in rCBF.

The investigation of brain perfusion in 5xFAD and TgCRND8 mice was performed by Tatryn et al. [59]. ASL-MRI was used to measure CBF, and dynamic susceptibility contrast-enhanced MRI was used to measure cerebral blood volume (CBV), while [^18^F]fluorodeoxyglucose (FDG) PET was used to measure brain metabolism. TgCRND8 displayed reduced perfusion in the cortex and hippocampus, as well as a reduced metabolism at 12 months of age, but no changes in the CBV were shown at an earlier age compared with controls. The 5xFAD mice showed a reduction in CBF at 7 months of age in the cortex but no difference at 12 months of age and increased CBV in the hippocampus and thalamus. In contrast, a different study on brain perfusion in 5xFAD mice at 11 months of age using [^99m^Tc]Tc-hexamethylpropyleneamine oxime (HMPAO) single-photon emission computed tomography (SPECT) highlighted no differences between transgenic and wild-type controls [60].

## 5. Histological Markers of Thrombosis in Patients with AD

There are a number of studies that show co-localisation of fibrinogen and Aβ plaques in brain samples from clinical studies. Increased abundance of fibrinogen chains α, β, and γ has been shown in the post-mortem brains of AD patients compared with controls [51], with chain β observed at the highest level [55]. Interestingly, Aβ deposits and fibrinogen have been shown to co-localise in the brain cortex [51,52,55,61] and hippocampus [55], and, in some cases, neurovascular occlusion has been shown [52,55]. Additionally, fibrinogen was also detected with Aβ, near blood vessels in places of synaptic dysfunction [55]. It has been shown that Dutch and Iowa Aβ mutations increase the affinity of Aβ to fibrinogen in CAA patients [61].

Additionally, the size of parenchymal vessels increased in AD brains compared with controls [52]. The number of large vessels (over 20 µm) that contained fibrinogen was quantified, and it was demonstrated that there were almost twice as many occluded vessels in AD brains compared with controls. It has been shown that patients diagnosed with atherosclerosis were more likely to have dementia and had lower scores on summary measures of cognitive domains ante mortem [62]. Atherosclerosis increased the risk of AD, which was shown to be proportional to the severity of the atherosclerosis diagnosis. The association was significant even when controlling for secondary factors (such as APOEε4 level, hypertension, diabetes, smoking, age, and infarcts).

Post-mortem analysis of AD patients showed platelet localisation in cerebral blood vessels [25], which was detected by immunostaining for platelet markers CD41 and CD62P. CD41-positive platelets were found to co-localise with Aβ, while CD62P+ platelets localised in blood vessels independently of Aβ plaques, with only limited distribution to the brain parenchyma. Accumulation of thrombin and prothrombin was also shown in post-mortem AD patients’ brain samples [63,64], specifically associated with Aβ plaques [63] and NFTs [63,64]. Thrombin levels have also been found to be elevated in the microvasculature [65,66] and cerebrospinal fluid [66] of AD patients compared with controls.

## 6. Platelet-Associated Markers in Patients with AD

An abnormal haemostatic state in dementia was initially thought to be involved only in VD and not in AD [67]. However, it has since been suggested that haemostasis perturbation may play a role in AD. Abnormal platelet activation and thrombosis biomarkers have been described in AD, although their full significance remains unclear. Increased platelet activation in AD patients has been documented multiple times [17,22,23,68]. It has been demonstrated that platelet activation, measured by the expression of the GPIIb-IIIa complex and P-selectin, could serve as a predictive biomarker for the level of cognitive decline in AD patients [17]. Furthermore, patients with pre-existing cardiovascular issues, such as coronary artery disease, are at a higher risk of developing AD [68]. In those patients, platelet activation measured by P-selectin expression was also associated with cognitive decline [68]. A similar relationship was observed for coated platelets, a subset of activated platelets stimulated by two agonists (collagen and thrombin), where a higher level of coated platelets was associated with a more rapid cognitive decline [22]. On the contrary, there are studies that have found that the mean platelet volume (a marker of platelet activation) decreased in AD patients [69]. Additionally, it has been shown that platelet function may act as a predictive biomarker for the later development of dementia. The Framingham Heart Study (FHS) is a longitudinal multi-generational study that began in 1948 and aims to assess the risk factors contributing to cardiovascular disease [70]. Data from the study indicate that patients who had a higher response to ADP at baseline were more likely to develop dementia [71]. Also, the membrane fluidity of platelets is impacted in AD patients, showing more fluidity in AD patients compared with controls [72].

Independently of platelet activity markers, the expression of different platelet proteins has been shown to be associated with and reliably predict AD. A study based on a combination of proteomics and traditional biochemistry highlighted upregulation of APP and increased secretion of Aβ_1–40_ in platelets from advanced AD patients [19]. The same study also identified key cytoskeletal proteins significantly altered in AD and mild cognitive impairment (MCI) patients, which included talin upregulated in mild AD patients, vinculin downregulated in mild AD patients, moesin downregulated in both mild and advanced AD patients, C3b downregulated in both MCI and advanced AD patients, and Rho upregulated in advanced AD patients. Notably, biochemical evidence of the disruption of glycogen synthase kinase 3-beta (GSK3B) homeostasis has been reported in platelets [73], where the proportion between phospho-GSK3B and total GSK3B (GSK3B ratio) is significantly reduced in patients with MCI and AD compared with controls (with a positive correlation with memory test results). A recent meta-analysis and systematic review from 2023, by Fu et al. [74], looked closely at platelet-associated biomarkers in AD patients. They analysed 88 clinical studies where platelet-associated biomarkers were investigated in over 700 AD patients (and a similar number of non-AD controls). Firstly, they reported that the platelet APP ratio (i.e., the percentage of 120–130 kDa to 110 kDa isoforms of the APP) is significantly decreased in the peripheral blood of AD patients compared with controls and proposed to utilise this parameter as a clinical biomarker for the diagnosis of AD. Further, they investigated the levels of platelet APP secretases, such as A-disintegrin, metalloprotease 10 (ADAM10), beta-site APP-cleaving enzyme 1 (BACE1), and presenilin-1 (PSEN-1). ADAM10 levels were decreased in platelets from AD patients vs. healthy controls, which correlated with the level of progression of AD. BACE1 seemed to be increased in AD, although statistical significance was not achieved. PSEN-1 levels were the same in the two cohorts. Nevertheless, combination of the above markers (platelet APP, ADAM1, BACE1, and PSEN1) was able to successfully diagnose AD with a sensitivity of 88.9%. In addition to Aβ protein pathway components being altered in AD, the tau protein ratio (high molecular weight/low molecular weight) was also increased in AD patients and was correlated with the level of cognitive decline. Platelet hyperactivity markers in AD patients are summarised in Table 2.

## 7. Hypercoagulation, Hyperlipidaemia, and Inflammation Markers in the Peripheral Blood of Patients with AD

Important plasma proteins involved in haemostasis have also been found to be elevated in AD, suggesting vascular involvement and a pro-thrombotic state in the disorder. Among these proteins, both activated factor VII and von Willebrand factor are elevated in AD patients [67]. Similarly, high levels of factor VIII [75] and plasminogen activator inhibitor-1 (PAI-1) have been associated with an increased risk of VD [75,76]. PAI-1 levels have been correlated with the level of neurological decline [76]. The relationship between plasma levels of fibrinogen and a higher risk for AD and VD has also been demonstrated [75,77]. Further, clot degradation products D-dimer and prothrombin fragment 1+2 levels correlated with more rapid cognitive decline [78]. It has also been shown that increased levels of D-dimer increase the risk of VD [79]. Higher levels of fibrinogen and D-dimer in VD were also found in a North-Indian population study, confirming the usefulness of homeostatic biomarkers in dementia diagnosis [80].

Park et al. [81] showed that levels of activated factor XII, activated factor XI, activated factor X, and prekallikrein in plasma are all increased in AD and correspond to AD progression. They also performed a receiver operating characteristic analysis to establish if any of the above factors could be used as a biomarker of AD. Using plasma levels of activated factor XII allowed them to distinguish between prodromal AD and AD patients from control samples, showing it could potentially be used as a biomarker of AD. Additionally, cerebrospinal fluid (CSF) levels of Aβ_1–42_ and plasma levels of the activated factor XII ratio could further increase the accuracy of staging the AD.

A Mendelian randomisation study by Shi et al. [82] studied the coagulation factor in AD. They used data from the following two different cohorts: UK Biobank and International Genomics of Alzheimer’s Project. They were able to find a causal link between higher levels of protein C, coagulation factor X, and activated partial thromboplastin time and a higher risk of developing AD. On the other hand, a high level of coagulation factor XI is causally associated with a lower risk of AD. Also, the authors did not find any link between AD and von Willebrand factor, coagulation factor VIII, PAI-1, ADAM with thrombospondin type 1 motif, member 13 (ADAMTS13), plasmin, endogenous thrombin potential, and coagulation factor VII; however, they do suggest more research and data are required on those factors.

Lipoprotein(a) is a known risk factor for atherosclerosis and related diseases, such as coronary heart disease and stroke [83], and its serum concentration correlates with the risk of AD [84] and VD [85,86]. Moreover, apolipoprotein(a) (small-molecular-weight isoform) has been shown to increase the risk of AD and VD [87]. Higher levels of human urotensin-II (UII) (a peptide involved in vasoconstriction) have also been associated with a higher risk of VD but not AD [86]. Other atherosclerotic markers, such as LDL [85,86], lipid peroxide [85,86], interleukin-6 (IL6) [86,88], and high-sensitivity C-reactive protein [86], have been shown to be increased in VD but not AD or controls. Nonetheless, increased levels of oxidised low-density lipoproteins, a known risk factor for atherosclerosis, did not increase the risk of developing dementia in a population-based longitudinal study (follow-up period: 2.2–10.3 years) [89]. Other biomarkers, such as serum levels of kallikrein 6, clusterin, and adiponectin, have been shown to be unchanged in dementia [88]. Matrix metalloproteinases (MMPs) have been found elevated in AD compared with healthy controls [90] and specifically in AD patients, higher levels of MMP2 in plasma (compared with VD) and MMP10 in CSF (compared with controls) [90]. A summary of coagulation-related markers in AD patients is presented in Table 3.

## 8. Clinical Imaging Evidence of Brain Hypoperfusion in Patients with AD

Johnson et al. [91] used ASL-MRI to measure CBF in a cohort of control, MCI, and dementia patients. They were able to show hypoperfusion regions in the brain in AD and MCI patients compared with controls [8]. Of interest, the reduced CBF areas were not always associated with grey-matter atrophy (measured by T1-weighted MRI) [8]. It has also been shown that perfusion [^99m^Tc]Tc-HMPAO SPECT imaging can be used to predict whether MCI patients will develop progressive MCI (PMCI), demonstrating a potential diagnostic value [92]. A longitudinal, three-year study on MCI and dementia patients showed differences in brain hypoperfusion in stable MCI compared with those who developed PMCI. The reduced perfusion in PMCI patients was similar to the changes in early AD patients. A different study has shown that a higher load of Aβ plaques (measured by PET [^18^F]florbetapir) correlated with lower CBF (measured by ASL-MRI) [93]. Finally, a longitudinal study utilising N-isopropyl-p-[^123^I] iodoamphetamine ([^123^I]IMP) to asses CBF in AD patients revealed that multiple vascular risk factors were associated with worse outcomes in both rCBF and cognition [94].

## 9. Conclusions and Future Perspectives

The accumulation of Aβ is a hallmark of AD. Although the effect of Aβ on neurons and its role in disease progression have been extensively researched and debated, the consequence of Aβ accumulation on cerebrovascular function has remained relatively unexplored. Abundant and convincing published evidence irrefutably associates AD with a pro-thrombotic state that is apparent in both animal models and patients. The evidence reported includes platelet hyperactivity in ex vivo functional and biochemical assays on platelets from animal models, histological evidence of neurovascular thrombosis in both mouse models and patients, and systemic markers of platelet hyperactivity and blood hypercoagulability in patients diagnosed with AD. Despite this overwhelming experimental evidence regarding the association of AD and thrombosis, the causative role of haemostasis dysregulation in AD onset and progression remains to be verified. This will be essential to consider haemostasis dysregulation and neurovascular thrombosis as valid interventional targets to treat or control AD. Considering the current lack of effective therapeutic tools to fight this disease and the increasing prevalence of AD in the population, a better understanding of the mechanisms underlying this disease is an important objective and an urgent endeavour for modern biomedical research. Crucially, research on the role of thrombosis in AD requires a significant refocus from observational to mechanistic studies, which may be facilitated by the development of novel and more effective experimental approaches. In summary, despite an increased research effort and a wealth of novel information on AD, the development of a treatment remains a challenge.

## Figures and Tables

**Table 1:** Studies showing platelet hyperactivity or hypercoagulation in mouse models of AD.

Mouse Model	Thrombosis Marker	Reference
Aged APP23	Increased platelet activation; Increased thrombi formation; Decreased clotting time in vivo.	Jarre et al. [24]
Middle-aged APP23	Decreased platelet activation; Increased number of dense granules; Decreased thrombus stability; Increased clotting time in vivo.	Donner et al. [49]
3xTg-AD	Increased platelet adhesion; Increased thrombus formation.	Canobbio et al. [50]
APP_SweDI	Platelet toxicity on healthy cerebrovascular and parenchyma in vitro.	Kniewallner et al. [18]
APP23	Fibrinogen polypeptide chains α, β, and γ co-localised with Aβ_40_.	Bian et al. [51]
TgCRND8	Fibrinogen co-localised with Aβ plaques; Decreased clearance of fibrinogen from brain.	Cortes-Cantelli et al. [52]
Tg6799	Better cognitive function with fibrinogen-inhibitor treatment.	Ahn et al. [53]

**Table 2:** Studies describing circulating platelet markers in patients with AD.

Post/Ante Mortem	Thrombosis Marker	Reference
Post mortem	Increased amount of fibrinogen polypeptide chains; Co-localisation of fibrinogen and Aβ in brain cortex and vessel walls.	Bian et al. [51]
Post mortem	Co-localisation of fibrinogen and Aβ in brain cortex and vessel walls; Occlusion of vessels with fibrinogen; Increased number of large vessels containing fibrinogen.	Cortes-Canteli et al. [52]
Post mortem	Increased amount of fibrin chain β; Co-localisation of fibrinogen and Aβ in brain cortex, hippocampus, and vessel walls; Occlusion of vessels with fibrinogen; Location of fibrinogen near synaptic disfunction places.	Cortes-Canteli et al. [55]
Post mortem	Co-localisation of fibrinogen and Aβ in brain cortex and vessel walls; Dutch and Iowa Aβ mutations, increase in the affinity of Aβ to fibrinogen in CAA patients	Cajamarca et al. [61]
Post mortem	Increased risk due to atherosclerosis and arteriolosclerosis.	Arvanitakis et al. [62]
Post mortem	Platelet localisation in brain vessels and Aβ plaques.	Kniewallner et al. [25]
Post mortem	Thrombin and prothrombin accumulation in Aβ plaques and NFT.	Akiyama et al. [63]
Post mortem	Thrombin and prothrombin accumulation in NFT.	Arai et al. [64]
Post mortem	Increased thrombin levels in the microvasculature and cerebrospinal fluid.	Grammas et al. [65]
Post mortem	Increased thrombin level in cerebrospinal fluid.	Yin et al. [66]
Ante mortem	Increased platelet activation.	Stellos et al. [17]
Ante mortem	Increased level of coated platelets	Prodan et al. [22]
Ante mortem	Decreased mean platelet volume.	Wang et al. [69]
Ante mortem	Increased response to ADP increases the risk of AD.	Ramos-Cejudo et al. [71]
Ante mortem	Increased platelet membrane fluidity.	Kozubski et al. [72]
Ante mortem	Increased APP expression in AD platelets; Increased platelet secretion of Aβ_1–40_ in advanced AD patients; Talin upregulated in platelets from mild AD patients; Vinculin downregulated in platelets from mild AD patients; Moesin downregulated in mild and advanced AD platelets; C3b downregulated in MCI and advanced AD patient platelets; Rho upregulated in platelets from advanced AD patients.	Gonzalez-Sanchez et al. [19]
Ante mortem	GSK3B ratio decreased in MCI and AD patient platelets (positive correlation with memory tests).	Forlenza et al. [73]
Ante mortem	Decreased level of APP ratio in AD platelets; Decreased level of ADAM10 in AD platelets; Increased level of tau protein ratio in AD platelets.	Fu et al. [74]

**Table 3:** Studies describing circulating coagulation markers in patients with AD.

Ante mortem	Increased levels of activated factor VII and von Willebrand factor.	Mari et al. [67]
Ante mortem	Increased levels of factor VIII and PAI-1 increase the risk of VD; Increased levels of fibrinogen increase the risk of VD.	Gallacher et al. [75]
Ante mortem	Increased level PAI-1 increases the risk of VD.	Oh et al. [76]
Ante mortem	Increased level of fibrinogen increases the risks of AD and VD.	van Oijen et al. [77]
Ante mortem	Increased D-dimer and prothrombin fragment 1 + 2 in AD.	Stott et al. [78]
Ante mortem	Increased level of D-dimer increases the risk of VD.	Carcaillon et al. [79]
Ante mortem	Increased level of D-dimer increases in VD; Increased level of fibrinogen in VD.	Vishnu et al. [80]
Ante mortem	Increased level of lipoprotein(a) increases the risk of AD.	Solfrizzi et al. [84]
Ante mortem	Increased levels of lipoprotein(a), LDL, and lipid peroxide in VD.	Watanabe et al. [85]
Ante mortem	Increased levels of lipoprotein(a), LDL, lipid peroxide, interleukin-6, and C-reactive protein increase the risk of VD; Higher levels of human UII increase the risk of VD.	Ban et al. [86]
Ante mortem	Increased level of apolipoprotein increases the risks of AD and VD.	Emanuele et al. [87]
Ante mortem	Increased level of IL6 in VD.	Dukic et al. [88]
Ante mortem	Increased level of oxLD does not increase the risks of AD or VD.	Murr et al. [89]
Ante mortem	Increased level of MMP2 in plasma in AD compared with VD. Increased level of MMP10 in CSF compared with controls.	Duits et al. [90]
Ante mortem	Increased levels of activated factor XII, activated factor XI, activated factor X, and prekallikrein in AD.	Park et al. [81]
Ante mortem	Increased levels of protein C, coagulation factor X, and activated partial thromboplastin time increase the risk of AD. Increased level of coagulation factor XI decreases the risk of AD. Von Willebrand factor, coagulation factor VIII, PAI-1, ADAM with thrombospondin type 1 motif, member 13 (ADAMTS13), plasmin, endogenous thrombin potential, and coagulation factor VII are not associated with higher or lower risk of AD.	Shi et al. [82]

## Data Availability

No new data were created or analyzed in this study. Data sharing is not applicable to this article.
